# Catalytic activation of hydrogen peroxide by Cr_2_AlC MAX phase under ultrasound waves for a treatment of water contaminated with organic pollutants

**DOI:** 10.1016/j.ultsonch.2023.106294

**Published:** 2023-01-07

**Authors:** Monireh Alimohamadi, Alireza Khataee, Samira Arefi-Oskoui, Behrouz Vahid, Yasin Orooji, Yeojoon Yoon

**Affiliations:** a Research Laboratory of Advanced Water and Wastewater Treatment Processes, Department of Applied Chemistry, Faculty of Chemistry, University of Tabriz, 51666-16471 Tabriz, Iran; b Рeoples’ Friendship University of Russia (RUDN University), 6 Miklukho-Maklaya Street, Moscow, 117198, Russian Federation; c Department of Chemical Industry, Technical and Vocational University (TVU), Tehran, Iran; d Department of Chemical Engineering, Tabriz Branch, Islamic Azad University, Tabriz, Iran; e College of Geography and Environmental Sciences, Zhejiang Normal University, 321004 Jinhua, China; f Department of Environmental and Energy Engineering, Yonsei University, Wonju, Republic of Korea

**Keywords:** Cr_2_AlC MAX phase, Sonocatalytic activation, Cr_2_AlC/H_2_O_2_/ultrasound, Organic pollutant, Degradation

## Abstract

This study aims to investigate the sonocatalytic activation of hydrogen peroxide (H_2_O_2_) using Cr_2_AlC MAX phase prepared by the reactive sintering process. The hexagonal structure of the crystalline MAX phase was confirmed by X-ray diffraction. Moreover, the compacted layered structure of the MAX phase was observed via scanning electron microscopy and high-resolution transmission electron microscopy. Under the desired operating conditions, Cr_2_AlC MAX phase (0.75 g/L) showed suitable potential to activate H_2_O_2_ (1 mmol/L) under sonication, thereby allowing a considerable removal efficiency for various organic pollutants, including dimethyl phthalate (69.1%), rifampin (94.5%), hydroxychloroquine (100%), and acid blue 7 (91.5%) with initial concentration of 15 mg/L within 120 min of treatment. Kinetic analysis proved that the degradation reaction followed pseudo-first-order kinetics. Scavenging tests demonstrated that hydroxyl radicals and singlet oxygen were effective species during degradation. Furthermore, a probable mechanism for dimethyl phthalate degradation was suggested according to gas chromatography-mass spectroscopy and nuclear magnetic resonance analyses. The obtained results confirmed the capability of the triple Cr_2_AlC/H_2_O_2_/US process as a promising method for treating contaminated water.

## Highlights

• Preparation and characterization of the Cr_2_AlC MAX phase.
• Efficient activation of H_2_O_2_ on Cr_2_AlC under ultrasonic irradiation.
• Degradation of dimethyl phthalate, rifampin, hydroxychloroquine, and acid blue 7.
• Association of radical and non-radical species in the degradation of organic pollutants.
• Suggesting degradation mechanism considering scavengers and intermediates.

## Graphical abstract

**Figure:**
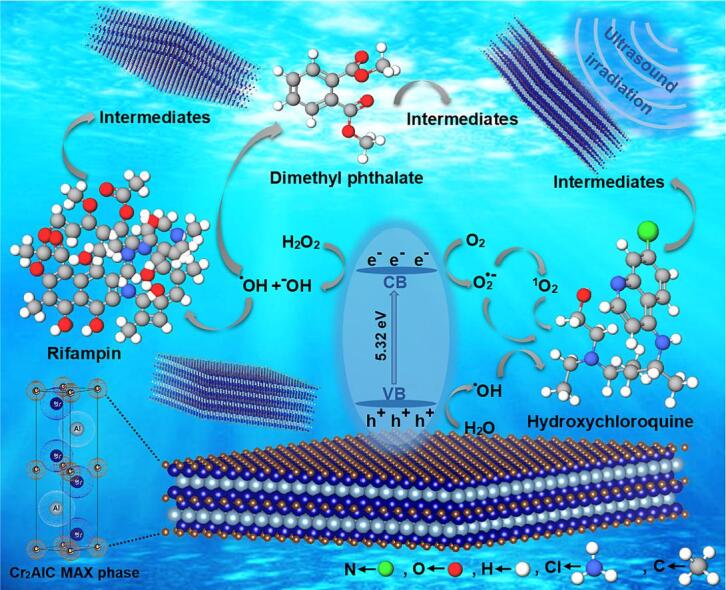

## 1 Introduction

MAX phases as newfangled nanolaminates are recently gaining interest in various applications owing to their desirable characteristics such as thermal and electrical conductivity, oxidation and corrosion resistance, strength and stiffness, elastic modulus, and lightweight nature [1]. For instance, their utilization has been reported for various industrial applications, such as in-core nuclear operations [2], fuel cladding construction of nuclear reactors [3], and photocatalytic processes [4]. MAX phases with three-dimensional structures have the general formula M_n+1_AX_n_, (n = 1–3), where ‘M’ represents a transition metal element (Sc, Ti, Zr, Hf, V, Nb, Ta, Cr, and Mo), ‘A’ represents an element from group 13 or 14 (Al, Si, P, S, Ga, Ge, As, In, Sn, Tl, and Pb), and ‘X’ represents nitrogen and/or carbon [5]. The chemical structures of the MAX phase present nanolayered stacking, with M−X octahedral layers divided by mono-atomic A-layers, which form the hexagonal *P*63/*mmc* cell [6], [7]. Based on their stoichiometry, MAX phases can be classified into three different phases based on the ‘n’ value including M_4_AX_3_ (4 1 3), M_3_AX_2_ (3 1 2), and M_2_AX (2 1 1) phases. Particularly, the Cr_2_AlC MAX phase as a member of M_2_AX phase, has a high oxidation resistance compared with that of other MAX phases like Ti_3_AlC_2_ or Ti_2_AlC. This makes it suitable for use in high-temperature applications [8].

Water contaminated with organic pollutants adversely affects human being’s health and is a critical global issue concerning environmental researchers [9]. Therefore, progress in water treatment techniques is a practical environmental priority. Advanced oxidation processes (AOPs), such as sonocatalysis, photocatalysis, sonophotocatalysis, ozonation, and Fenton and photo-Fenton reactions, are promising techniques owing to their high removal efficiency, biocompatibility, cost effectiveness, production of non-toxic compounds, and possibility of allowing reactions at ambient pressure and temperature [10], [11], [12]. During sonication, ultrasound (US) waves passing from an aqueous media can cause the breakage of chemical bonds and consequent free radical production [13], [14], [15]. The use of US alone to obtain a high removal efficiency for organic contaminants usually requires a large amount of energy owing to the considerable energy loss during this process. Thus, a catalyst that provides nucleation sites for cavitation can be added to the solution to overcome this obstacle, thereby resulting in a greater generation of active radicals [14]. Moreover, the US-assisted catalytic activation of oxidants like hydrogen peroxide (H_2_O_2_) is based on the heightened generation of reactive species, particularly hydroxyl radicals (^•^OH). Notably, H_2_O_2_ is considered an environmentally friendly oxidant in green chemistry and its activation results in the production of both radical ($O_{2}^{\cdot -}$ and ^•^OH) and non-radical (^1^$O_{2}$) species, which can be applied to degrade various pollutants such as dyes, drugs, phthalate esters, and other pollutants [16], [17], [18], [19].

Phthalate esters are additives and plasticizers, which are widely employed as dispersants, lubricants, binders, stabilizers, film formers, or gelling and emulsifying agents [20]. Dimethyl phthalate (DMP) is a common phthalate ester, which is used to intensify the mechanical properties and flexibility of products. Due to the lack of chemical bonding with the other molecules, it can be easily separated from plastic products and moved into the environment [21], [22]. Based on the results reported in Guangzhou city in south China, DMP existed in most water samples [23]. Furthermore, the concentration of DMP in landfill leachate was reported about 300 mg/L in Europe [24]. It is a nonbiodegradable contaminant, which has been placed in priority environmental pollutant lists owing to its toxicity, carcinogenesis, endocrine disrupting effects, continual bioaccumulation, and long hydrolysis half-life [25], [26], [27]. Therefore, to save human health and the water environment, developing efficient methods such as AOPs is critical for the DMP treatment from water resources.

In the present study, a Cr_2_AlC MAX phase was prepared via the reactive sintering procedure, and characterized through X-ray diffraction (XRD), scanning electron microscopy (SEM), high-resolution transmission electron microscopy (HRTEM), energy-dispersive X-ray spectroscopy (EDS), dot mapping, X-ray photoelectron spectroscopy (XPS), Fourier-transform infrared spectroscopy (FT-IR), ultraviolet–visible diffuse reflectance spectroscopy (UV–Vis DRS), and Brunauer–Emmett–Teller (BET) analyses. Then, the sonocatalytic activation of H_2_O_2_ was studied using the Cr_2_AlC MAX phase for DMP degradation in water. The effect of the major operating parameters, such as catalyst dosage, oxidant concentration, pH, and pollutant initial concentration was investigated and the synergy factor was evaluated for the triple Cr_2_AlC/H_2_O_2_/US system based on degradation kinetics. Afterwards, the performance of the Cr_2_AlC/H_2_O_2_/US process for degrading diverse organic pollutants, including hydroxychloroquine (HCQ), rifampin (RIF), and acid blue 7 (AB7), was assessed under the identified optimal operating conditions. Furthermore, scavenging tests were implemented to recognize the contribution of radical and non-radical species within the degradation process. A plausible treatment mechanism was suggested according to gas chromatography-mass spectroscopy (GC–MS) and nuclear magnetic resonance (NMR) analyses. To the best of our knowledge, this study is the first attempt to determine the catalytic function of the Cr_2_AlC MAX phase for the degradation of organic contaminants using the Cr_2_AlC/H_2_O_2_/US method.

## 2 Materials and methods

### 2.1 Materials

Graphite powder (3200 mesh, 99%) was supplied by Aladdin Reagent Co., ltd. (China). Chromium and aluminum powders (99.95%) with a 200 mesh size were purchased from SCRC of National Medicine Group (China). Moreover, H_2_O_2_, DMP (C_10_H_10_O_4_), sodium hydroxide (NaOH), hydrochloric acid (HCl), sodium nitrate (NaNO_3_), ethanol (C_2_H_5_OH), l*-*histidine (C_8_H_13_N_3_O_4_), *tert*-butanol (C_4_H_10_O), ethylenediaminetetraacetic acid (EDTA, C_10_H_16_N_2_O_8_), and o-phenylenediamine (C_6_H_4_(NH_2_)_2_) were supplied by Merck Co. (Germany). Additionally, HCQ (C_18_H_26_ClN_3_O), RIF (C_43_H_58_N_4_O_12_), and AB7 (C_37_H_35_N_2_Na O_6_S_2_) were supplied by Mofid Pharmaceutical Co. (Iran), Hakim Pharmaceutical Co. (Iran), and Shimi Boyakhsaz Co. (Iran), respectively.

### 2.2 Preparation of Cr_2_AlC MAX phase

The modified reactive sintering procedure was employed to prepare the Cr_2_AlC MAX phase inspired by the literature [28], [29]. Briefly, chromium, aluminum, and graphite powders were mixed in a planetary ball mill system (350 rpm, 18 h, 5 mm diameter zirconia balls in absolute ethanol). The molar ratio of Cr:Al:C and ball:material ratio were 2:1:1 and 10:1, respectively. The resultant powder was sintered via a conventional nonlinear hot-press protocol (1400 ℃, 20 MPa) under an inert atmosphere [30]. Subsequent to cooling, the obtained disk was crushed and sifted to obtain the Cr_2_AlC MAX phase powder [31].

### 2.3 Analytical methods

The Cr_2_AlC MAX phase powder was analyzed for structural identification using an X-ray diffractometer (SmartLab, Japan) including Cu Kα radiation (40 kV, 100 mA). Furthermore, SEM micrographs, EDS spectrum, and dot mapping patterns were recorded using the Tescan Mira3 microscope (Czech Republic) for analyzing the surface morphology and elemental characterization, respectively. Additionally, HRTEM images were obtained by a JEM-2100 Plus electron microscope (JEOL, Japan) to further assess the surface morphology. The surface compound and oxidation states of the prepared MAX phase were studied by XPS carried out by a Thermo Scientific Escalab 250Xi Plus XPS spectrometer (UK). Surface functional groups were identified by FT-IR spectrum recorded on a Bruker Tensor 27 spectrometer (Germany). The N_2_ adsorption/desorption analysis was performed using Belsorp Mini II (Japan, 77 K) to determine the specific surface area of the MAX phase by BET method. UV–Vis DRS was acquired to investigate the optical properties of the Cr_2_AlC MAX phase with an UV–Vis spectrophotometer (PerkinElmer, USA) while utilizing the reflectance standard of barium sulfate. The ^1^H NMR signals of DMP and its degradation intermediates were determined by a Bruker-Spectrospin 400 MHz UltraShield^TM^ NMR spectrometer. Agilent 6890 gas chromatography supported by an Agilent 5973 mass spectrometer (Palo Alto, CA) was applied to analyze the intermediates formed during the oxidation of DMP. Total organic carbon (TOC) measurements were carried out by Shimadzu TOC analyzer (Japan).

### 2.4 Experimental procedure

In this study, DMP degradation was implemented in an ultrasonic bath (Ultra-8060, JPL company, England) operating at a power and frequency of 150 W and 36 kHz, respectively, at 20℃. In a typical run, a defined dose of Cr_2_AlC MAX phase powder (0.75 g/L) was added to DMP solution (15 mg/L, 100 mL) in a 250 mL Pyrex glass flask. The H_2_O_2_ addition at the first step can affect the degradation efficiency sharply owing to its effect on the hydroxyl radicals production. To observe the effect of H_2_O_2_ addition in every step it was decided to add H_2_O_2_ at 10 min intervals. Experiments were carried out at the original pH of the DMP solution (8). The reaction bulk was exposed to sonication and the solution (3 mL) was withdrawn and filtered from the catalyst through a 0.22 µm syringe filter at specific time intervals to measure the concentration of DMP using a UV–Vis spectrophotometer (Specord 250, Analytik Jena, Germany) at the maximum wavelength of 277 nm. The effect of various parameters such as catalyst dosage, oxidant concentration, pH, pollutant initial concentration, and radical quenching agents were studied. For evaluating the reusability, the sample was washed three times with distilled water and dried after usage. The initial pH of the solution was adjusted using HCl (0.1 mol/L) and NaOH (0.1 mol/L) solutions.

### 2.5 Determination of pH_pzc_

To determine pH_pzc_ of the Cr_2_AlC MAX phase using the pH drift method [32], [33], five NaNO_3_ solutions with a concentration of 0.01 mol/L were prepared and their pH levels were adjusted to 2, 4, 6, 8, 10 using HCl and NaOH solutions. Then, 0.075 g of the catalyst was added to the solutions and all obtained suspensions were stirred in a shaker at the speed of 200 rpm and the temperature of 20 °C for 24 h. Ultimately, the final pH of the solution was plotted versus the initial pH. The point of zero charge (pH_pzc_) of the Cr_2_AlC MAX phase was determined from the point at which the initial pH equals the final pH.

## 3 Results and discussion

### 3.1 Catalyst characterization

**Fig. 1:**
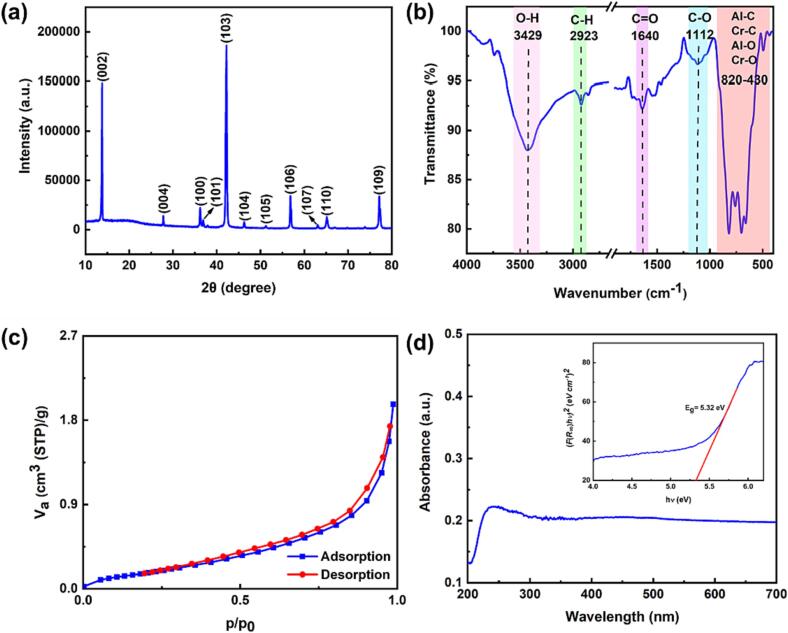
(a) XRD pattern, (b) FT-IR spectrum, (c) N_2_ adsorption/desorption isotherm, and (d) DRS diagram and Tauc plot (insert) of Cr_2_AlC MAX phase.

The XRD pattern of the as-prepared Cr_2_AlC MAX phase is presented in Fig. 1a. The noticeable sharp peaks verify the formation of Cr_2_AlC MAX phase. Moreover, the observed peaks at 2θ = 14.1°, 28.0°, 36.4°, 37.0°, 42.2°, 46.4°, 51.5°, 57.0°, 63.2°, 65.4°, and 77.3° corresponded to the (0 0 2), (0 0 4), (1 0 0), (1 0 1), (1 0 3), (1 0 4), (1 0 5), (1 0 6), (1 0 7), (1 1 0), and (1 0 9) planes of the so-synthesized sample, respectively, which demonstrate the hexagonal structure of the prepared MAX phase [34], [35], [36]. Additionally, the mean crystallite size of the MAX phase was appointed as 143 nm from the intense XRD peak (2θ = 42.2°) using the Debye–Scherrer equation [5], [37].

FT-IR analysis was conducted to demonstrate the surface functional groups in Cr_2_AlC MAX phase (Fig. 1b). The peaks appeared around 430–820 cm^−1^ and showed the stretching vibration modes of Al-O and Cr-O bonds and the presence of Cr-C and Al-C bonds [5], with the peaks detected at 1112, 1640, and 2923 cm^−1^ being ascribed to C—O, C=O, and C—H bonds, respectively, which are formed due to the oxidation of end-groups [38], [39]. The peak at 3429 cm^−1^ was related to the –OH stretching bond, confirming the presence of water molecules on the MAX phase surface [40], [41].

The N_2_ adsorption/desorption isotherm is presented in Fig. 1c. A standard reversible type III isotherm was recognized, proving that the Cr_2_AlC MAX phase was a nonporous material. The specific surface area (S_BET_) of the Cr_2_AlC MAX phase was calculated as 0.75326 m^2^/g by the multipoint BET method [8]. The optical properties of the Cr_2_AlC MAX phase were studied via the UV–Vis DRS method (Fig. 1d). The Kubelka–Munk function (Eqs. (1 and 2)) was employed to determine the band-gap energy of the MAX phase [5], [42], [43], [44].

(1) $$F\left(R_{\infty }\right)=\;\frac{{\left(1-\;R_{\infty }\right)}^{2}}{2R_{\infty }}$$

(2) $${\left(\alpha hv\right)}^{2}=C\;\left(\mathrm{hv}-E_{g}\right)\;$$

where F is the Kubelka–Munk function and R_∞_ refers to diffuse reflectance; and E_g_, *v*, *h*, and C are the optical bang gap, frequency of light, Planck’s constant, and the equation constant, respectively. The prepared MAX phase had a band gap energy of 5.32 eV, obtained by extrapolating the linear portion of ${\left(F\;\left(R_{\infty \;}\right)h\Delta \right)}^{2}$ vs energy (hѵ); the studies which reported that sonocatalytic processes can produce sonoluminescence with an energy of 6 eV [45], [46], confirming that the Cr_2_AlC MAX phase containing wide band gap can be activated under sonication to generate the electron–hole pairs [19], [47]. Moreover, the valence band (VB) and conduction band (CB) potentials of Cr_2_AlC were determined using Eqs. (3–5).

(3) $$E_{\mathrm{vB}}=X\;-E_{e}\;+\;\frac{E_{g}}{2}$$

(4) $$E_{\mathrm{CB}}=\;E_{\mathrm{VB}}-\;E_{g}$$

(5) $$X={\left[X_{\left(A\right)}^{a}\;X_{\left(B\right)}^{b}\;X_{\left(C\right)}^{c}\right]}^{1/\left(a+b+c\right)}$$

where E_e_ is the energy of an electron vs energy of hydrogen (4.5 eV), X corresponds to the absolute electronegativity, and E*_VB_* and E*_CB_* represent the valence band and conduction band potentials, respectively. Further, a, b, and c refer to the amount of each constituent in the sample [5], [44]. Consequently, the values of VB and CB for the Cr_2_AlC MAX phase were calculated to be −0.01 eV and −5.33 eV, respectively.

**Fig. 2:**
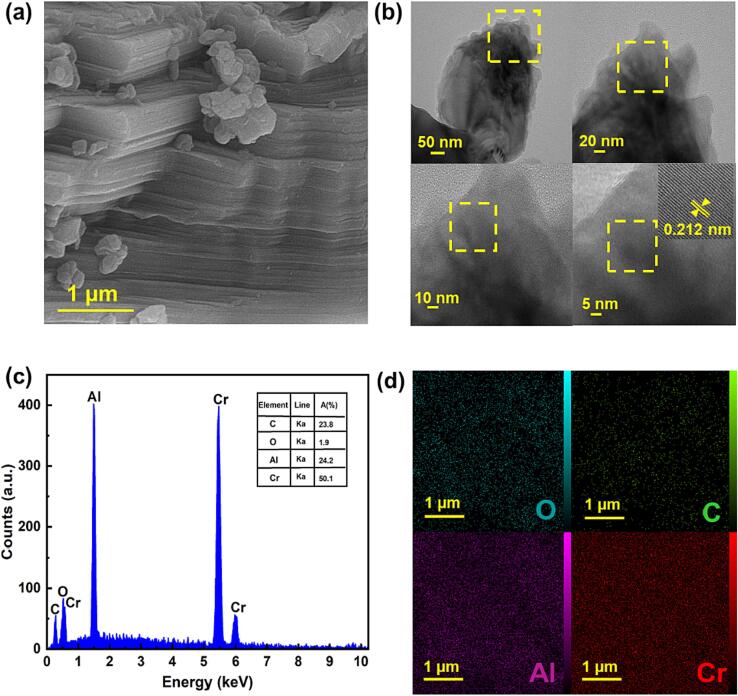
(a) SEM image, (b) HRTEM images, (c) EDS spectrum, and (d) elemental dot mapping of Cr, Al, O and C for the Cr_2_AlC MAX phase.

The compressed layered morphology of Cr_2_AlC MAX phase can be observed in SEM image (Fig. 2a), which is in agreement with the morphologies reported in similar studies [34], [48]. HRTEM images also clarified the layered structure of the Cr_2_AlC MAX phase (Fig. 2b). Specifically, the lattice with d space of the 0.212 nm observed in HRTEM image can be assigned to the (1 0 3) plane of Cr_2_AlC MAX phase in 2θ = 42.2°, which is in well-supported by the XRD results [34]. Furthermore, the existence of Cr, Al, O, and C without other elements in the MAX phase was also identified via EDS and elemental dot mapping; the atomic percentage was reported inside of the EDS spectrum. as can be seen the atomic percentage relatively corresponds to the formula of Cr_2_AlC MAX phase (Fig. 2 (c and d)).

**Fig. 3:**
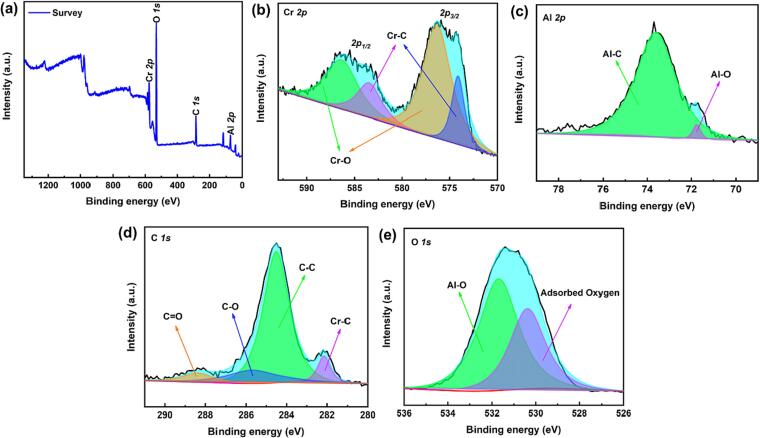
XPS spectra of (a) survey, (b) Cr 2p, (c) Al 2p, (d) C 1 s, and (e) O 1 s for CrAl_2_C MAX phase.

To study the surface composition and valence states of the Cr_2_AlC MAX phase, XPS analysis of the structural elements was carried out; as depicted in Fig. 3a, the existence of Cr, Al, O, and C elements was proven, consistent with previous studies [49], [50]. Considering the Cr *2p* core levels of Cr_2_AlC, it included two doublets: Cr *2p_3/2_* and Cr *2p_1/2_*. Two peaks at binding energies of 583.6 and 574.3 eV corresponded to Cr-C, and the other two peaks at binding energies of 586.5 and 576.3 eV could be ascribed to Cr-O. This indicates that the MAX phase powder was exposed to the air, thereby causing surface oxidation and the production of surface termination O groups (Fig. 3b). Al *2p* peaks were observed at 73.6 and 71.8 eV (Fig. 3c), related to Al-C and Al-O bonds, respectively. High-resolution C *1 s* spectrum identified binding energies of 288.4, 285.7, 284.5, and 282.2 eV (Fig. 3d) corresponding to C=O, C—O, C—C, and Cr-C bonds, respectively. The O *1 s* spectrum is depicted in Fig. 3e; the peak placed at 531.7 eV corresponded to the Al-O bond. Moreover, the second peak placed at 530.4 eV demonstrated oxygen bonding and indicated oxygen adsorption on the MAX phase surface. Thus, the XPS results indicated the production of a high-purity Cr_2_AlC MAX phase.

### 3.2 Sonocatalytic activation

**Fig. 4:**
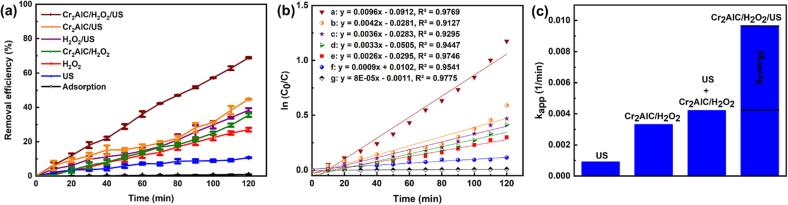
(a) Removal efficiency of DMP in different processes, (b) pseudo-first-order kinetic rate plot for different processes, and (c) determination of synergy factor for the Cr_2_AlC/H_2_O_2_/US process (operating conditions: [Cr_2_AlC] = 0.75 g/L, [H_2_O_2_] = 1 mmol/L, [DMP] = 15 mg/L and pH = 8).

The removal efficiency of DMP was monitored in various systems, including adsorption, US, H_2_O_2_, Cr_2_AlC/H_2_O_2_, H_2_O_2_/US, Cr_2_AlC/US, and Cr_2_AlC/H_2_O_2_/US, under the same operating conditions within 120 min of reaction time (Fig. 4a). As can be observed, DMP showed insignificant adsorption (0.9%) on the surface of the MAX phase, which can be attributed to the repulsive effect of surface charges and the low specific surface area of the Cr_2_AlC MAX phase. Low removal efficiencies observed for US and H_2_O_2_ systems (10.8% and 25.8%, respectively) demonstrated that these systems could not sufficiently remove DMP [46]. The Cr_2_AlC/H_2_O_2_, H_2_O_2_/US, and Cr_2_AlC/US processes displayed removal efficiencies of 33.9%, 37.5%, and 44.6%, respectively. The coupled systems could thus enhance the treatment of organic contaminants [51]. However, the Cr_2_AlC/H_2_O_2_/US process showed desired performance for DMP treatment (69.1%). Each degradation processes in different systems were repeated in the same operating condition three times and their error bars were calculated. As can be seen in Fig. 4a, each data point and error bar represent the mean and the standard deviations of independent triplicates, respectively. A kinetics study for DMP removal elucidated that all processes followed pseudo-first order kinetics, with correlation coefficient (R^2^) values higher than 0.9 (Fig. 4b), which is in agreement with the results of other studies [52]. Moreover, for better interpretation, the synergy factor for the Cr_2_AlC/H_2_O_2_/US process was obtained 2.3 using Eq. (6) based on an apparent pseudo-first-order reaction rate constant (k_app_) (Fig. 4c) [19].

(6) $$\text{Synergy factor}=\frac{{\text{k}}_{\text{app catalyst}/{\text{H}}_{2}{\text{O}}_{2}/\text{US}}}{{\text{k}}_{\text{app US}}+{\text{k}}_{\text{app catalyst}/{\text{H}}_{2}{\text{O}}_{2}}}\;$$

Improved DMP removal for the Cr_2_AlC/H_2_O_2_/US process could be explained through the possible reactions mentioned in Eqs. (7–11) [53], [54].

(7) $$Cr_{2}AlC+US\to Cr_{2}AlC\;\left(h_{\mathrm{VB}}^{+}+e_{\mathrm{CB}}^{-}\right)$$

(8) H2O+hVB+→O·H+H+

(9) H2O2+eCB-+US→O·H+OH-

(10) $$O_{2}+e_{\mathrm{CB}}^{-}\to O_{2}^{\cdot -}$$

(11) 2O2·-+2H2O→O21+H2O+2OH-

Based on Eq. (7), the excitement of the Cr_2_AlC MAX phase under the US irradiation generated the electron–hole pairs; as indicated in Eq. (8), ^•^OH could be produced through the oxidation of H_2_O by the formed holes. Furthermore, the produced electrons could react with H_2_O_2_ and O_2_, thereby leading to the production of hydroxyl and superoxide radicals, respectively (Eqs. (9 and 10)). Additionally, the reaction between superoxide radicals and water molecules produced singlet oxygen (^1^$O_{2}$) (Eq. (11)). Consequently, the active species generated by the excited catalyst under the US waves in the presence of H_2_O_2_ improved the removal efficiency of DMP. In addition, the cavitation bubble formation was enhanced during sonication owing to the low tensile strength between the solid–liquid surface and nucleation sites in the catalyst, which improved the overall removal efficiency [19]. Consequently, the triple Cr_2_AlC/H_2_O_2_/US process was selected for conducting further runs.

### 3.3 Effects of operational parameters on removal efficiency and reusability of catalyst

**Fig. 5:**
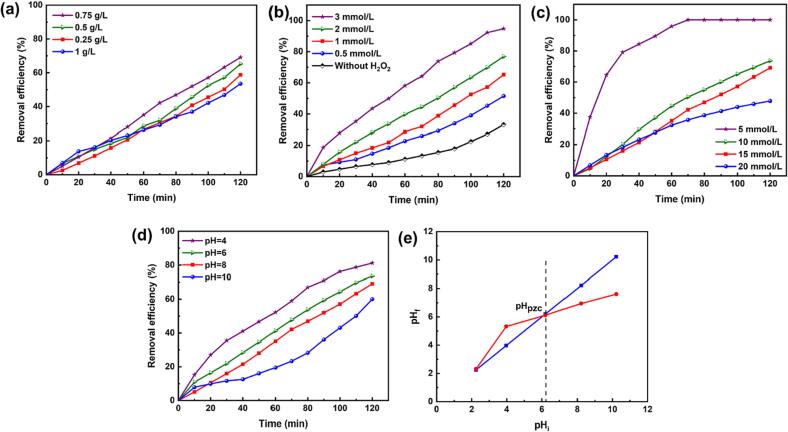
Effect of (a) catalyst dosage, (b) H_2_O_2_ concentration, (c) DMP concentration, and (d) initial pH on DMP removal efficiency; and (e) pH_pzc_ in Cr_2_AlC /H_2_O_2_/US process (operating conditions: [Cr_2_AlC] = 0.75 g/L, [H_2_O_2_] = 1 mmol/L, [DMP] = 15 mg/L, and pH = 8).

The effects of the main operational parameters, including catalyst dosage, pH, oxidant concentration, and pollutant initial concentration, on the removal of DMP were studied for the Cr_2_AlC/H_2_O_2_/US process. As shown in Fig. 5a, when the catalyst amount increased from 0.25 to 0.75 g/L, the removal efficiency of DMP improved; whereas a decline in the removal efficiency was identified when using 1 g/L of catalyst, which was ascribed to its effect on preventing US waves from penetrating the solution and to the accumulation of solid particles [5], [55]. The removal efficiency increases with an increase in the H_2_O_2_ concentration, which favors the enhanced generation of ^•^OH (Fig. 5b) [56]. According to Fig. 5c, an increase in the initial DMP concentration leads to a decrease in the removal efficiency; this can be ascribed to the agglomeration of DMP on active sites, which restricts energy absorption by the catalyst, thereby decreasing the production of oxidizing species. Moreover, there are more pollutants and the degradation intermediates that have to be degraded by the process [57]. The results of DMP degradation at different pH levels (4–10) are presented in Fig. 5d, wherein the removal efficiency increased in an acidic medium. Based on the results reported in Fig. 5e, the pH_pzc_ was determined to be 6.2 which is in agreement with the literature reporting pH_pzc_ of the carbon based MAX phases around 6–7 [47], [58]. The pollutant has a nonionic form and the MAX phase surface is positively charged when the pH is lower than pH_pzc_, which results in electrostatic attraction with the electron-rich aromatic nuclei or oxygen in DMP [20], [55]. In contrast, the catalyst surface has negative charge when the pH is higher than pH_pzc_, and this decreases DMP adsorption. Furthermore, when the pH is higher than 8, carbon dioxide can be generated and transformed to $CO_{3}^{2-}$, which can consume ^•^OH [20]; consequently, the removal efficiency decreases. Furthermore, the effect of the solution pH on the adsorption of DMP on the MAX phase surface was evaluated in the range of 4–8. Based on the results reported in Fig. S1, the adsorption of the pollutant on the MAX phase increased in the acidic medium which can be attributed to the electrostatic attraction between the positively charged MAX phase and electron-rich aromatic nuclei or oxygen in DMP. The effect of different catalysts on the degradation of DMP was compared with the present work and summarized in Table. S1.

**Fig. 6:**
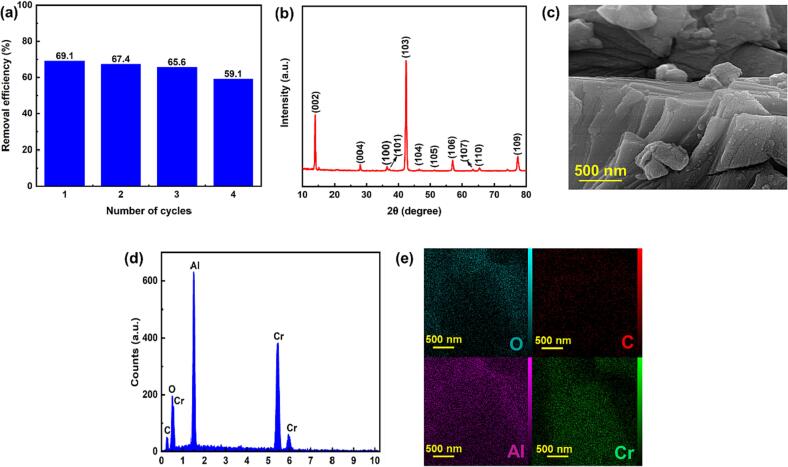
(a) Reusability of Cr_2_AlC MAX phase, (b) XRD pattern, (c) SEM image, (d) EDS spectrum, and (e) elemental dot mapping of Cr, Al, O, and C for reused CrAl_2_C MAX phase over four cycles of DMP removal. (operating conditions: [Cr_2_AlC] = 0.75 g/L, [H_2_O_2_] = 1 mmol/L, [DMP] = 15 mg/L, and pH = 8).

The reusability of a catalyst is an important parameter for its practical application. Therefore, four recycling runs were carried out under the desired condition. A slight change in the removal efficiency was observed (Fig. 6a), suggesting the stable performance of the MAX phase with consecutive usage. Moreover, the structure of the reused catalyst after four experiments were checked using XRD, SEM, EDS, and elemental dot mapping analyses (Fig. 6 (b–e)); the results revealed that the characteristics of the used sample were the same as those of the as-prepared sample confirming the structural stability of the MAX phase in Cr_2_AlC /H_2_O_2_/US process [5].

### 3.4 Removal of various organic contaminants and predominant oxidizing species

**Fig. 7:**
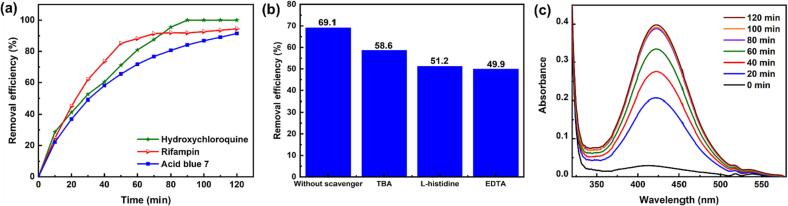
(a) Removal of different organic contaminants, (b) effect of various scavengers on DMP removal efficiency with molar ratios of TBA and l-histidine/DMP = 80, and EDTA/DMP = 40, and (c) spectra of OPD-trapped ^•^OH in the Cr_2_AlC /H_2_O_2_/US process (operating conditions: [Cr_2_AlC] = 0.75 g/L, [H_2_O_2_] = 1 mmol/L, [DMP] = 15 mg/L, and pH = 8).

The removal of different organic pollutants, such as HCQ, RIF, and AB7, was investigated via the Cr_2_AlC/H_2_O_2_/US process under the desired operational parameters. HCQ is a drug that is commonly used for malaria prophylaxis and treatment. This drug has anti-inflammatory and antiviral effects, and is also used for the treatment of a wide range of chronic diseases such as the 2019 novel coronavirus (COVID-19) [59]. Moreover, RIF as an antimicrobial antibiotic and a promising pharmaceutical used for treating different infections such as tuberculosis [60]. In addition, AB7 as a triarylmethane dye is widely employed in textile industries; it is a recalcitrant anionic dye with a chemically stable structure, long chains, and high molecular weight [61]. All mentioned compounds can penetrate various aqueous sources owing to their solubility and stability. As depicted in Fig. 7a, a remarkable removal efficiency was acquired for HCQ (100%), RIF (94.5%), and AB7 (91.5%) after 120 min, which confirms the potential of the triple Cr_2_AlC/H_2_O_2_/US process for industrial wastewater treatment.

Scavengers are widely used to quench the free active species that participate in the removal of organic pollutants. Their role in the Cr_2_AlC/H_2_O_2_/US process was evaluated using various scavengers, including *tert*-butanol (TBA), l-histidine, and EDTA which were applied to suppress ^•^OH, ^1^$O_{2}$, and hole respectively. The effect of the scavengers on the degradation efficiency of DMP using triple Cr_2_AlC/H_2_O_2_/US process was reported in Fig. 7b. As depicted, the removal efficiency decreased in the presence of TBA, thereby indicating the role of ^•^OH in DMP degradation [62], [63]. Additionally, a decreased removal efficiency was noted in the presence of l-histidine, indicating the role of ^1^$O_{2}$ during the treatment process [64]. Significantly, decrement in the removal efficiency in presence of EDTA affirmed the role of holes in DMP degradation [65]. Further decreases in the removal efficiency of DMP by the l-histidine scavenger compared with TBA revealed the high contribution of ^1^$O_{2}$ in the DMP degradation process. Moreover, the formation of ^•^OH during the triple process in the absence of DMP was probed using o-phenylenediamine (OPD) [66]. The reaction between OPD and ^•^OH yielded 2,3-diaminophenazine (λ_max_ = 419 nm) (Eq. (12)), was distinguished using the UV–Vis spectrophotometer. The results shown in Fig. 7c prove the generation of ^•^OH during the process.(12)
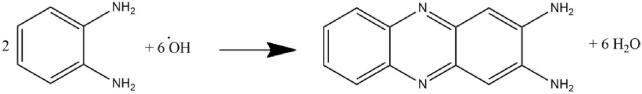


**Fig. 8:**
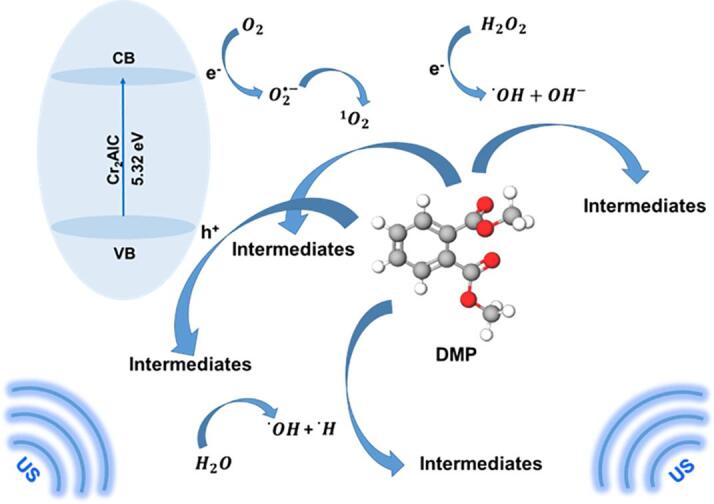
Degradation of DMP by the Cr_2_AlC/H_2_O_2_/US process.

Consequently, the results confirmed that both radical and nonradical species play a role in DMP degradation (Fig. 8). $O_{2}$ and H_2_O_2_ could be converted to ^1^$O_{2}$ and ^•^OH, respectively, on the catalyst surface (Eqs. (9–11)) according to the conduction band value .

### 3.5 GC–MS and NMR analyses for identifying possible degradation pathways

The GC–MS method was implemented to identify possible pathways for DMP degradation. The molecular structure and analytical properties of identified intermediates are presented in Table 1. Five possible intermediates, namely benzene, methyl acetate, acetic acid, carbonic acid, and 2-butanol, were recognized during the degradation process. Hence, the degradation may involve C—C partitioning, elimination reactions, and reactive radical addition [67]. Moreover, two plausible pathways are suggested: (i) breakage in the main structure of DMP and formation of the simplest aromatic structure (benzene); and (ii) production of hydroxylated compounds owing to the electrophilic O—H group addition to the divided aliphatic part of DMP produces methyl acetate, acetic acid, carbonic acid, and 2-butanol. Finally, the byproducts generated during the removal reactions can be further mineralized into CO_2_ and H_2_O. Additionally, some inorganic compounds may have been produced during the removal process, but not detected because of their limited retention time in the GC–MS. Moreover, TOC removal of 53.3% was obtained for pollutant solution containing 15 mg/L of DMP and 0.75 g/L catalysts within 360 min of the reaction time, confirming the mineralization of DMP by the triple Cr_2_AlC/H_2_O_2_/US process.

**Table 1:** Byproducts generated during DMP degradation process.

No.	Compound names	Structure	Tg(min)	Main fragments(m/z) (percent)
1	Benzene		34.953	207.00 (100.00%), 73.10 (60.03%), 55.00 (44.15%), 281.00 (41.37%), 57.10 (39.28%)
2	Methyl acetate		4.138	116.10 (100.00%), 75.10 (99.63%), 73.10 (15.17%), 117.10 (12.81%), 76.10 (9.82%)
3	Acetic acid		4.06	75.10 (100.00%), 116.10 (98.91%), 73.10 (15.93%), 117.00 (13.09%), 76.00 (10.43%)
4	Carbonic acid		3.583	75.00 (100.00%), 116.10 (89.45%), 73.10 (14.20%), 117.00 (11.19%), 76.00 (9.69%)
5	2-Butanol		3.095	59.00 (100.00%), 147.00 (38.36%), 75.00 (14.83%), 141.10 (8.43%), 73.00 (8.38%)

**Fig. 9:**
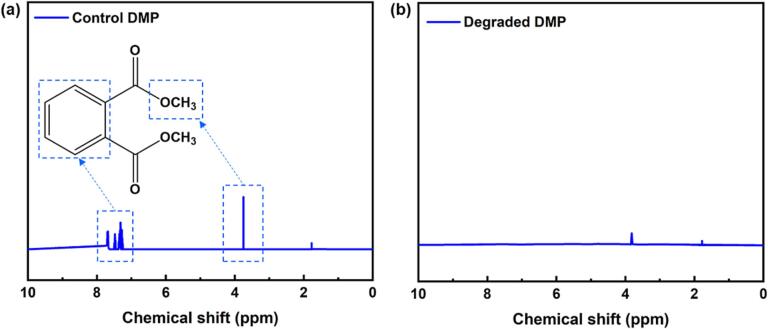
(a and b) Comparative ^1^H NMR spectra of control and degraded DMP samples in D_2_O solvent (operating conditions: [Cr_2_AlC] = 0.75 g/L, [H_2_O_2_] = 1 mmol/L, [DMP] = 15 mg/L, pH = 8, ^1^H NMR range = 0–10 ppm, number of scans = 5, and solvent = D_2_O).

The NMR investigation also confirmed the degradation of DMP via the triple Cr_2_AlC/H_2_O_2_/US system [68]. For ^1^H NMR analysis, a sample was dried (50 °C) and held under vacuum, and ^1^H NMR spectra were recorded in D_2_O as a solvent. Fig. 9 (a and b) demonstrate comparative ^1^H NMR spectra for the control and degraded DMP, respectively. The signals of aromatic protons for the control DMP were observed in the 6–8 ppm region. After 100% degradation of DMP during a long time process, significant peaks for the aromatic region disappeared, thereby confirming the destruction of the stable aromatic ring. Proton peaks in the shielded region remained in the aliphatic region. This demonstrates the existence of small-chained hydrocarbons produced during the degradation procedure, which are well-supported with the GC–MS results.

## 4 Conclusion

In this investigation, Cr_2_AlC MAX phase powder was prepared by the reactive sintering method. The desired characteristics of the hexagonal MAX phase, with high purity, were confirmed by diverse characterization analyses. The XRD pattern confirmed the high crystallinity of the prepared MAX phase. Furthermore, compacted layered morphology of the MAX phase was observed in SEM and HRTEM images. Surface functional groups, oxidation states, and elemental compositions of the MAX phase were assessed by FT-IR, XPS, and EDS analyses. The catalytic activation of H_2_O_2_ by the MAX phase under US waves was evaluated for degrading 15 mg/L DMP. Under the obtained desired conditions, the activation of 1 mmol/L H_2_O_2_ by 0.75 g/L Cr_2_AlC MAX phase under US irradiation within 120 min showed the significant removal of organic pollutants, including DMP (69.1%), HCQ (100%), RIF (94.5%), and AB7 (91.5%). The scavenging tests revealed that ^•^OH and ^1^$O_{2}$ were the oxidizing species and that ^1^$O_{2}$ was the predominate reactive agent. In addition, generated holes play important role in the degradation of organic pollutant. Moreover, GC–MS and NMR analyses identified a possible mechanism for DMP degradation. The observed results confirmed that the combined Cr_2_AlC/H_2_O_2_/US process is promising for the degradation of different contaminants in the field of water and wastewater treatment.

## CRediT authorship contribution statement

**Monireh Alimohamadi:** Investigation, Visualization, Writing – original draft. **Alireza Khataee:** Supervision, Writing – review & editing. **Samira Arefi-Oskoui:** Conceptualization, Writing – review & editing. **Behrouz Vahid:** Writing – review & editing. **Yasin Orooji:** Writing – review & editing. **Yeojoon Yoon:** Writing – review & editing.

## Declaration of Competing Interest

The authors declare that they have no known competing financial interests or personal relationships that could have appeared to influence the work reported in this paper.

## Data Availability

The data that has been used is confidential.
