# Supplementary material for: Catalytic activation of hydrogen peroxide by Cr2AlC MAX phase under ultrasound waves for a treatment of water contaminated with organic pollutants
Source: Ultrason Sonochem. 2023 Jan 7;93:106294. doi: 10.1016/j.ultsonch.2023.106294 (PMC9852641; doi:10.1016/j.ultsonch.2023.106294)
Supplement: Supplementary data 1 [file mmc1.docx]

**Supplementary information**

**Catalytic activation of hydrogen peroxide by Cr_2_AlC MAX phase under ultrasound waves for a treatment of water contaminated with organic pollutants**


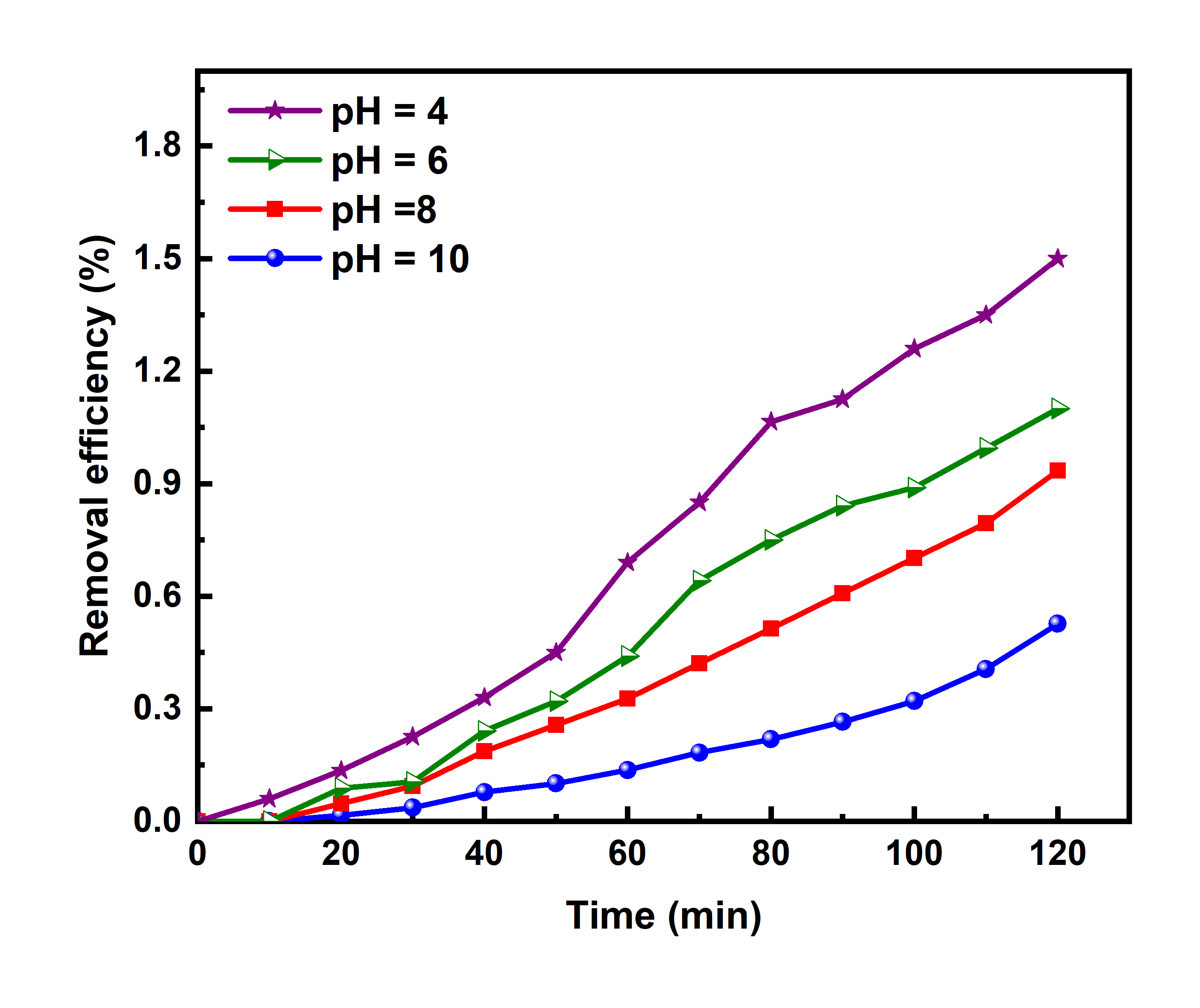


**Fig. S1.** Effect of initial solution pH on the adsorption of DMP on the surface of Cr_2_AlC MAX phase (operating conditions: [Cr_2_AlC] = 0.75 g/L, and [DMP] = 15 mg/L).

**Table. S1.** Comparison of different catalysts for DMP degradation.

| Ref. | Removal efficiency (%) | Time (min) | Operating conditions | Pollutant concentration | Catalyst dosage | AOP method |
| --- | --- | --- | --- | --- | --- | --- |
| [1] | 65 | 120 | pH = 3.0,  [PMS] = 2 mmol/L | DMP (2 mg/L) | 0.5 g/L | g-C_3_ N_4_/PMS/UV |
| [2] | ~25 | 120 | pH = 5 | DMP (20 mg/L) | 1 g/L | La-TiO_2_/UV |
| [3] | 62 | 60 | pH= 6.8,  [PMS] = 1.2 mmol/L | DMP (10 mg/L) | 0.15 g/L | CoFe-LDH/PMS |
| [4] | ~80 | 120 | US power = 150 W | DMP (40µg/L) | - | US |
| [5] | ~65 | 120 | UV power = 450 W | DMP (33.3 mg/L) | 10g/L | TiO_2_/UV |
| [6] | ~40 | 30 | pH = 6.8 | DMP (20 mg/L) | 0.3 g/L | Fe_3_O_4_/MWCNTs |
| [7] | 100 | 100 | US power = 400 W,  [H_2_O_2_] = 0.1 mol/L | Rif (30 mg/L) | 1 g/L | ZnO/GO/H_2_O_2_/US |
| [8] | 83 | 90 | US power = 300 W,  pH = 5 | Rif (20 mg/L) | 1.5 g/L | ZrO_2_-tuf/US |
| [9] | 100 | 120 | US power = 150 W, | Rif (10 mg/L) | 1 g/L | Ti_2_SnC/US |
| [10] | 76.44 | 60 | UV power = 150 W,  [S_2_O_8_^2-^]= 6 mmol/L,  pH= 5 | AB7 (10 mg/L) | 6 mg/L | MOF-2/S_2_O_8_^2-^/US |
| [11] | 70.8 | 60 | UV power = 150 W,  [PDS]= 6 mmol/L,  pH= 8.3 | AB7 (10 mg/L) | 2 mg/L | Zif-8/PDS/US |
| [9] | 95 | 120 | US power = 150 W | AB7 (10 mg/L) | 1 g/L | Ti_2_SnC/US |
| [12] | 70 | 120 | US power = 150 W,  pH = 8.7 | HCQ (20 mg/L) | 0.1 g/L | MoS_2_/CNTs/US |
| [13] | 60.42 | 80 | US power = 150 W,  [PDS]= 15 mmol/L | HCQ (20 mg/L) | 0.2 g/L | Ti_3_GeC_2_/PDS/US |
| [14] | 98 | 120 | pH = 7 | HCQ (5 mg/L) | 0.15 g/L | Kaolin |
| Present study | 69.1  94.5  91.5  100 | 120 | UV power = 150 W,  pH=8,  [H_2_O_2_] = 1 mmol/L | DMP (15 mg/L)  Rif (15 mg/L)  AB7 (15 mg/L)  HCQ (15 mg/L) | 0.75 g/L | Cr_2_AlC/H_2_O_2_/US |

**Reference:**

[1] L. Xu, L. Qi, Y. Sun, H. Gong, Y. Chen, C. Pei, L. Gan, Mechanistic studies on peroxymonosulfate activation by g-C_3_N_4_ under visible light for enhanced oxidation of light-inert dimethyl phthalate, Chinese Journal of Catalysis. 41 (2020) 322–332. https://doi.org/10.1016/S1872-2067(19)63447-9.

[2] Y. Chen, M. Ran, Z. Zhou, X. Han, H. Zhu, J. Gu, Lanthanum/titanium dioxide immobilized onto industrial waste with enhanced photocatalytic activity, and the degradation of dimethyl phthalate, Journal of Cleaner Production. 321 (2021) 129014. https://doi.org/10.1016/j.jclepro.2021.129014.

[3] Q. Ye, J. Wu, P. Wu, S. Rehman, Z. Ahmed, N. Zhu, Enhancing peroxymonosulfate activation by Co-Fe layered double hydroxide catalysts via compositing with biochar, Chemical Engineering Journal. 417 (2021) 129111. https://doi.org/10.1016/j.cej.2021.129111.

[4] E. Psillakis, D. Mantzavinos, N. Kalogerakis, Monitoring the sonochemical degradation of phthalate esters in water using solid-phase microextraction, Chemosphere. 54 (2004) 849–857. https://doi.org/10.1016/j.chemosphere.2003.09.039.

[5] D. Balabanič, D. Hermosilla, N. Merayo, A.K. Klemeničič, Á. Blanco, Comparison of different wastewater treatments for removal of selected endocrine-disruptors from paper mill wastewaters, The Journal of Environmental Science and Health, Part A, Toxic/Hazardous Substances and Environmental Engineering. 47 (2012) 1350–1363. https://doi.org/10.1080/10934529.2012.672301.

[6] Z. Bai, Q. Yang, J. Wang, Catalytic ozonation of dimethyl phthalate using Fe_3_O_4_/multi-wall carbon nanotubes, Environmental Technology. (United Kingdom). 38 (2017) 2048–2057. https://doi.org/10.1080/09593330.2016.1245360.

[7] Ā. Afroozān Bāzghale, A. Mohammad-Khāh, Improvement of Ultrasound-Assisted Removal of Rifampin in the Presence of N: ZnO/GO Nanocomposite as Sonocatalyst, ChemistrySelect. 5 (2020) 4413–4421. https://doi.org/10.1002/slct.202000068.

[8] A. Khataee, P. Gholami, B. Kayan, D. Kalderis, L. Dinpazhoh, S. Akay, Synthesis of ZrO_2_ nanoparticles on pumice and tuff for sonocatalytic degradation of rifampin, Ultrasonics Sonochemistry. 48 (2018) 349–361. https://doi.org/10.1016/J.ULTSONCH.2018.05.008.

[9] S. Haddadi, A. Khataee, S. Arefi-oskoui, B. Vahid, Y. Orooji, Ultrasonics Sonochemistry Titanium-based MAX-phase with sonocatalytic activity for degradation of oxytetracycline antibiotic, Ultrasonics Sonochemistry. 92 (2023) 106255. https://doi.org/10.1016/j.ultsonch.2022.106255.

[10] A.J. Sisi, A. Khataee, M. Fathinia, B. Vahid, Ultrasonic-assisted degradation of a triarylmethane dye using combined peroxydisulfate and MOF-2 catalyst: Synergistic effect and role of oxidative species, Journal of Molecular Liquids. 297 (2020) 111838. https://doi.org/10.1016/J.MOLLIQ.2019.111838.

[11] A. Jamal Sisi, M. Fathinia, A. Khataee, Y. Orooji, Systematic activation of potassium peroxydisulfate with ZIF-8 via sono-assisted catalytic process: Mechanism and ecotoxicological analysis, Journal of Molecular Liquids. 308 (2020) 113018. https://doi.org/10.1016/j.molliq.2020.113018.

[12] M. Dastborhan, A. Khataee, S. Arefi-Oskoui, Y. Yoon, Synthesis of flower-like MoS_2_/CNTs nanocomposite as an efficient catalyst for the sonocatalytic degradation of hydroxychloroquine, Ultrasonics Sonochemistry. 87 (2022) 106058. https://doi.org/10.1016/j.ultsonch.2022.106058.

[13] Z. Ansarian, A. Khataee, S. Arefi-Oskoui, Y. Orooji, H. Lin, Ultrasound-assisted catalytic activation of peroxydisulfate on Ti_3_GeC_2_ MAX phase for efficient removal of hazardous pollutants, Materials Today Chemistry. 24 (2022) 100818. https://doi.org/10.1016/J.MTCHEM.2022.100818.

[14] H. Bendjeffal, M. Ziati, A. Aloui, H. Mamine, T. Metidji, A. Djebli, Y. Bouhedja, Adsorption and removal of hydroxychloroquine from aqueous media using Algerian kaolin: Full factorial optimisation, kinetic, thermodynamic, and equilibrium studies,  International Journal of Environmental Analytical Chemistry. 00 (2021) 1–22. https://doi.org/10.1080/03067319.2021.1887162.
